# Amyotrophic Lateral Sclerosis and Multiple Sclerosis Overlap: A Case Report

**DOI:** 10.1155/2012/324685

**Published:** 2012-12-24

**Authors:** Francesca Trojsi, Anna Sagnelli, Giovanni Cirillo, Giovanni Piccirillo, Cinzia Femiano, Francesco Izzo, Maria Rosaria Monsurrò, Gioacchino Tedeschi

**Affiliations:** ^1^Department of Neurology, Second University of Naples, 80138 Naples, Italy; ^2^Magnetic Resonance Imaging Research Center SUN-FISM, Neurological Institute for Diagnosis and Care “Hermitage Capodimonte”, 80131 Naples, Italy

## Abstract

The concurrence of amyotrophic lateral sclerosis (ALS) and multiple sclerosis (MS) is extremely rare. We reported the case of a 33-year-old woman with a past history of paresthesias at the right hand, who developed progressive quadriparesis with muscular atrophy of limbs and, finally, bulbar signs and dyspnea. Clinical and neurophysiologic investigations revealed upper and lower motor neuron signs in the bulbar region and extremities, suggesting the diagnosis of ALS. Moreover, magnetic resonance imaging (MRI) and cerebrospinal fluid (CSF) analysis demonstrated 3 periventricular and juxtacortical lesions, hyperintense in T2 and FLAIR sequences, and 3 liquoral immunoglobulin G (IgG) oligoclonal bands, consistent with diagnosis of primary progressive MS (PPMS). This unusual overlap of ALS and MS leads to the discussion of a hypothetical common pathological process of immunological dysfunction in these two disorders, although the role of immune response in ALS remains ambivalent and unclear.

## 1. Introduction

Amyotrophic lateral sclerosis (ALS) is a neurodegenerative disorder, characterized by progressive weakness of limb, bulbar, and respiratory muscles, with an annual incidence in Europe of 2.16/100.000 person years [1]. Multiple sclerosis (MS), an inflammatory disease of the central nervous system (CNS) with dissemination of demyelinating lesions in time and space, mainly affects young adults and has a variable incidence by racial heritage and geographic region (0.8 to 12/100.000 person years) [2]. Thus, the concurrence of these two neurological disorders is extremely infrequent [3–6], although recent explanatory studies have suggested a possible familial aggregation between MS and ALS [7, 8]. 

We report the clinical and radiological findings of a young woman with the unusual combination of ALS and MS, also reviewing the limited literature on the coexistence of these two disorders in the same patient. 

## 2. Case Report

Our patient was a 33-year-old woman, who noted persistent paresthesias and progressive weakness at the right hand, significantly decreased after three months without requiring therapy. One year later, she experienced worsening of weakness and appearance of atrophy at the right arm, which gradually extended at the left arm and lower limbs. After eight months, dysphagia, dysarthria, and dyspnea occurred. 

Her familial and past medical history was not indicative of pathologic conditions. The patient comes from Lacedonia, in the South of Italy near Avellino, and her family came from the nearby town of Belvedere Marittima for several generations. 

Neurological examination revealed dysphagia and dysarthria, with mild tongue atrophy, spastic quadriparesis, brisk upper and lower extremity reflexes, pyramidal signs, and widespread atrophies of the limbs muscles. The sensory exam was normal. The laboratory exams were all normal. Specifically, we performed serum dosage of anti-nucleus, anti-DNA and anti-cardiolipin antibodies and both serum and cerebrospinal fluid (CSF) dosage of anti-gangliosides GM1, anti-viral (hepatitis virus B and C, Herpesviridae, *Morbillivirus*, *Rubivirus*, *Enterovirus*, adenovirus, paramyxovirus, respiratory syncytial virus, and Retroviridae-human T-lymphotropic virus-1 or HTLV-1 and human immunodeficiency virus or HIV) and anti-Borrelia antibodies.

Electromyography (EMG) showed pathological spontaneous activity at rest (fibrillations and fasciculations) and chronic, neurogenic motor unit changes in three sites (bulbar, upper and lower limbs), whilst motor and sensory nerve conductions were normal and conduction blocks were not detected. 

**Figure 1:**
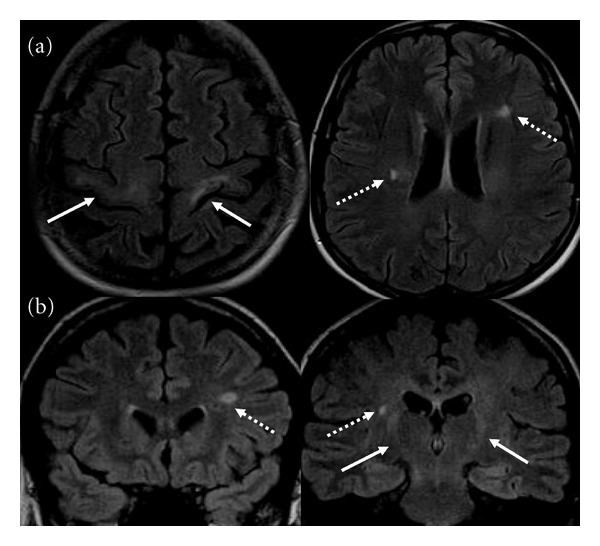
Axial (a) and coronal (b) FLAIR MR images showing typical hyperintensities of pyramidal tracts (solid line), from motor cortices to bulbar pyramids. In this context, periventricular and juxtacortical lesions, hyperintense in T2 and FLAIR (dashed line), were observed.

Brain MRI showed bilaterally T2 and fluid attenuate inversion recovery (FLAIR) hyperintensities of pyramidal tracts and precentral cortexes, together with 3 periventricular or juxtacortical white matter lesions, hyperintense in T2 and FLAIR sequences (Figure 1), and gadolinium enhancement of the lesion in the right corona radiata. No abnormal areas were evident in the spinal cord. These MRI scans were performed at 1.5 Tesla, about two years after the first symptoms onset (i.e., when the patient came to our observation for the first time). To exclude possible coexistent tumor or inflammatory conditions, the patient underwent a whole body 18-fluorodeoxyglucose-positron emission tomography (FDG-PET) study six months later, which did not identify areas of abnormal cellular metabolism, and also brain and spinal MRI scans, which did not show novel pathological areas. Afterwards, no other MRI exam has been performed because of the progressive impairment of the respiratory function of the patient.

The cerebrospinal fluid (CSF) analysis detected an increased immunoglobulin G (IgG) or Link index (0.8) and 3 IgG oligoclonal bands (OCBs) by isoelectric focusing, without evidence of barrier injury. The CSF was negative for viral and Lyme antibodies. 

We screened genetic susceptibility to motor neuron disorders by testing some genes linked to similar phenotypes of ALS in the Italian population: we did not found any mutation of superoxide dismutase 1 (SOD1), transactive response-DNA binding protein (TARDBP), fusion in malignant liposarcoma/translocated in liposarcoma (FUS/TLS) and C9ORF72 genes. 

We assumed that in this remarkable case the diagnostic criteria for both ALS [9] and primary progressive MS (PPMS) [10] were fulfilled.

The patient was treated with riluzole (50 mg × two/die) and physiotherapy. She is receiving artificial respiratory support (noninvasive ventilation) from about six months after the diagnosis of ALS because of the worsening of respiratory function (forced vital capacity or FVC < 50%).

## 3. Discussion

We report clinical and instrumental findings about an intriguing case of overlap between ALS and MS. Specifically, besides the diagnostic criteria for ALS [9], in this case the 2010 McDonald criteria for diagnosis of MS with an insidious neurological progression (PPMS) were also met [10]. In fact, our patient presented at least one year of insidious neurological progression, in addition to (i) evidence for dissemination of brain lesions in space (DIS), based on ≥1 T2 lesions in the MS-characteristic (periventricular, juxtacortical, or infratentorial) regions, and (ii) positive CSF analysis (isoelectric focusing evidence of OCBs and/or elevated IgG index).

In particular, in our case the clinical course of the two concomitant neurological disorders evokes the previous reports of Dynes et al. [3] and Li et al. [4] who observed the neuropathological coexistence and the simultaneous clinical progression of both ALS and MS. Interestingly, they have been identified as key pathological features degeneration of pyramidal tracts and anterior horns cells in both cervical and lumbar cord in addition to multiple demyelinating plaques in prototypic locations (i.e., cortex, periventricular region, corpus callosum, brainstem, and spinal cord). Undoubtedly, a relevant limitation of our report is the lack of an autoptic confirmation of ALS and MS coexistence, although clinical and instrumental findings are suggestive of this overlap of neurological diseases in our patient.

About the detection of CSF OCBs in our case, it is remarkable that an intrathecal synthesis of IgG may be observed also in patients with ALS alone, although in a small subset (approximately 0.5–3.5%) [11, 12]. Interestingly, in a recent study by Ticozzi et al. [12], CSF was collected from 259 patients with ALS, screened also for mutations of SOD1, FUS, TARDBP, angiogenin (ANG), optineurin (OPTN), and C9ORF72 genes. It was found that, among patients with OCBs, two patients had the TARDBP p.A382T mutation (one of which in homozygous state), and one the ANG p.P-4S variant. These results prompted to hypothesize that mutations in both TARDBP and ANG genes may have induced damage of the blood-brain barrier (BBB), promoting local immune responses and neuroinflammation.

Other than the above-mentioned pathologic descriptions, among the rare clinical reports of patients with both ALS and MS, Hewitt et al. [5] observed the coexistence of MS and familial ALS (FALS) in a patient with mutation of FUS/TLS gene (p.Gly174del), and, more recently, Ismail et al. [6] identified the C9ORF72 expansion in 80% of patients with MS-ALS among a large population of ALS patients from the North of England. Remarkably, further recent evidences give clues about the genetic similarities that the two diseases share. In fact, it has been revealed that first degree relatives of MS patients are significantly more prone to ALS and vice versa [7, 8]. 

Interestingly, in patients with MS lower motor neuron dysfunction has been occasionally reported, particularly with impairment of motor functions of the hands and sometimes associated with MRI detection of cervical plaques [13, 14]. In our report, no evidence of demyelinating plaques in the spinal cord was observed. However, our longitudinal MRI examination was limited in time because of the onset of respiratory failure which made difficult to execute follow-up MRI scans. 

Although we do not aim to suggest any causative relationship between ALS and MS, we hypothesize that this association might probably be induced by some common pathological mechanisms of interplay between inflammation and degeneration in both disorders. In fact, several neurological diseases, including ALS and MS, are currently associated with chronic neuroinflammation [4, 6, 15]. Despite the prominent role of the autoimmunity in the pathogenesis of MS, the manifold characteristics of immune response in ALS are beginning to be appreciated, although they remain largely unclear [6, 15]. 

## 4. Conclusions

We assume that our unusual report, in addition to the limited literature on the concurrence of ALS and MS, might be useful to stimulate further analyses on the unknown physiopathological aspects of these two disorders, even to shed light on the role of the immune response in the pathogenesis of ALS.
